# Profiling the Murine SUMO Proteome in Response to Cardiac Ischemia and Reperfusion Injury

**DOI:** 10.3390/molecules25235571

**Published:** 2020-11-27

**Authors:** Paul W. Hotz, Marion Wiesnet, Georg Tascher, Thomas Braun, Stefan Müller, Luca Mendler

**Affiliations:** 1 Institute of Biochemistry II, Goethe University Medical School, University Hospital Building 75, Theodor-Stern-Kai 7, 60590 Frankfurt am Main, Germany; hotz@stud.uni-frankfurt.de (P.W.H.); tascher@med.uni-frankfurt.de (G.T.); 2 Department of Cardiac Development and Remodeling, Max Planck Institute for Heart and Lung Research, Ludwigstrasse 43, 61231 Bad Nauheim, Germany; Marion.Wiesnet@mpi-bn.mpg.de (M.W.); Thomas.Braun@mpi-bn.mpg.de (T.B.)

**Keywords:** cardiac I/R injury, SUMO, SENP, immunoprecipitation, mass spectrometry, proteomics

## Abstract

SUMOylation is a reversible posttranslational modification pathway catalyzing the conjugation of small ubiquitin-related modifier (SUMO) proteins to lysine residues of distinct target proteins. SUMOylation modifies a wide variety of cellular regulators thereby affecting a multitude of key processes in a highly dynamic manner. The SUMOylation pathway displays a hallmark in cellular stress-adaption, such as heat or redox stress. It has been proposed that enhanced cellular SUMOylation protects the brain during ischemia, however, little is known about the specific regulation of the SUMO system and the potential target proteins during cardiac ischemia and reperfusion injury (I/R). By applying left anterior descending (LAD) coronary artery ligation and reperfusion in mice, we detect dynamic changes in the overall cellular SUMOylation pattern correlating with decreased SUMO deconjugase activity during I/R injury. Further, unbiased system-wide quantitative SUMO-proteomics identified a sub-group of SUMO targets exhibiting significant alterations in response to cardiac I/R. Notably, transcription factors that control hypoxia- and angiogenesis-related gene expression programs, exhibit altered SUMOylation during ischemic stress adaptation. Moreover, several components of the ubiquitin proteasome system undergo dynamic changes in SUMO conjugation during cardiac I/R suggesting an involvement of SUMO signaling in protein quality control and proteostasis in the ischemic heart. Altogether, our study reveals regulated candidate SUMO target proteins in the mouse heart, which might be important in coping with hypoxic/proteotoxic stress during cardiac I/R injury.

## 1. Introduction

Acute coronary occlusion is a leading cause of morbidity and mortality in western countries according to the world health organization [1]. Revascularisation and reperfusion are the current state of the art treatments for patients, however, this can induce further deterioration of the myocardium known as ischemia/reperfusion (I/R) injury [2,3]. Much progress has been made to identify the underlying molecular mechanisms associated with cardiac I/R injury [4,5], nevertheless, very few new drugs were derived from such efforts. Accordingly, there is an urgent need to develop new therapeutic approaches for effective cardioprotection during ischemia and reperfusion.

Post-translational modification (PTM) of proteins with small ubiquitin-like modifiers (SUMOs) controls important cellular pathways and functions as a powerful regulatory switch in entire tissues, including also the developing and adult heart [6,7]. In mammalian cells, SUMO is expressed as an immature precursor [8]. After the C-terminal diglycine motif is exposed via proteolytic processing by SUMO specific isopeptidases (SENPs) [8,9,10], SUMO forms get conjugated to lysine residues of proteins through an enzymatic cascade (E1, E2, E3) in an ATP dependent process. SUMO1 shares about 50% amino acid sequence identity with SUMO2 and SUMO3, which are distinguished just by a few amino acids [11]. Like ubiquitin, SUMO, in particular SUMO2/3, can also form polymeric chains on its substrates [8]. Chain formation can prime substrates for subsequent ubiquitination and proteasomal degradation by SUMO-targeted ubiquitin ligases, such as RNF4 and RNF111 [12,13,14]. In general, SUMOylation modifies hundreds of mainly nuclear, but also extra-nuclear proteins and is thereby able to alter protein-protein interactions, protein activities or their cellular distribution in a highly dynamic manner. The best characterized deSUMOylating enzymes belong to the SENP family. In humans, six different SENPs are known (SENP1-3, SENP5-7), which all share a conserved catalytic domain, consisting of a typical cysteine protease catalytic triad [8,9,15]. While SENP1 and SENP2 cleave both SUMO1 and SUMO2/3 conjugates, SENP3, 5, 6 and 7 are mainly specific to SUMO2/3 with SENP6/7 preferentially trimming SUMO chains [8,9].

The important role of the SUMO system in cardiac development and the pathomechanism of heart failure has been demonstrated by cardiac specific genetic modulation of the SUMO machinery (i.e., SUMOs, conjugation enzymes and SENPs) [7], but the role of SUMOylation in cardiac I/R injury has remained largely elusive [16,17,18,19,20].

Whereas previous studies concentrated mainly on the altered pattern of SUMOylation of individual proteins in the heart, the SUMO conjugation/deconjugation machinery coordinates signaling networks by targeting groups of protein functioning in common signaling pathways. Here, we aimed at analyzing the SUMO system in response to cardiac ischemia and reperfusion by performing an unbiased system-wide SUMO proteome approach in vivo to gain a global overview about the role of SUMOylation in the molecular regulation of I/R injury.

Here we provide evidence that the general SUMOylation pattern of proteins is dynamically regulated in the heart with decreased SENP activities throughout ischemia and reperfusion. Moreover, we identify several SUMO1 and SUMO2 target proteins in vivo, with a sub-group of proteins showing a regulated pattern during I/R injury, e.g., hypoxia- or angiogenesis related transcription factors and proteasome subunits which might be important in coping with hypoxic and proteotoxic stress during cardiac I/R damage.

## 2. Results and Discussion

### 2.1. Cardiac Ischemia Reduces Global Protein SUMOylation and Decreases the Amount and Catalytic Activity of deSUMOylating Enzymes

**Figure 1:**
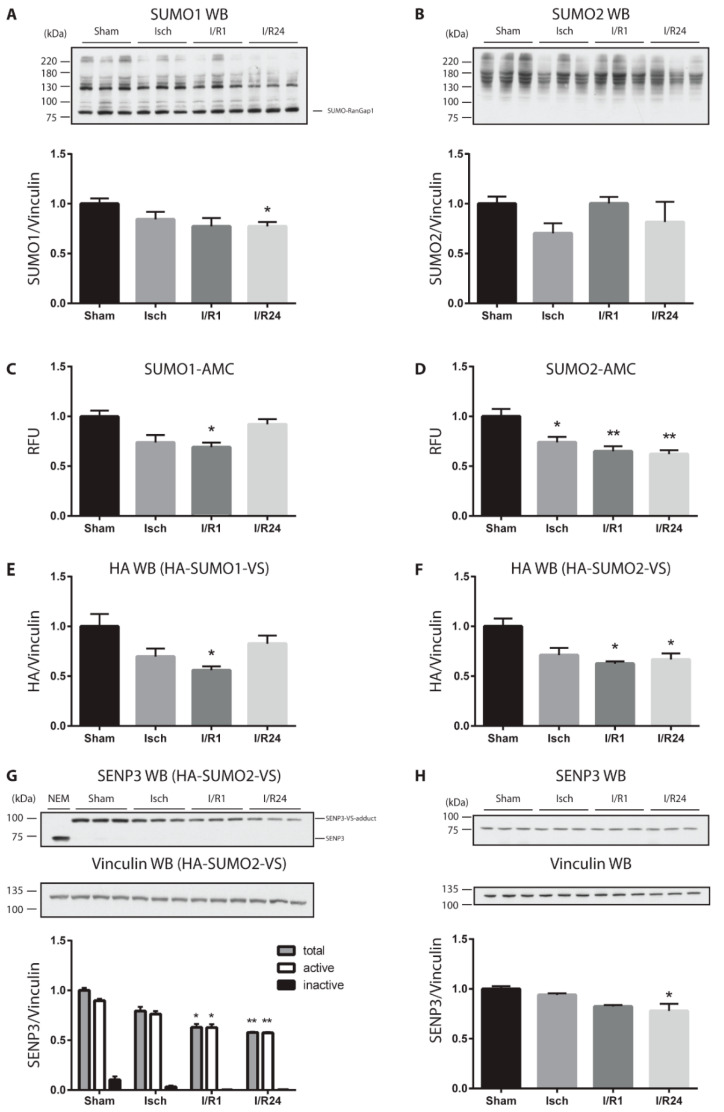
SUMOylation/DeSUMOylation changes dynamically during ischemia and reperfusion in the mouse heart. (**A**,**B**) Whole homogenates (1% SDS buffer) from Sham, ischemic (Isch), and ischemic/reperfused (I/R, reperfusion time 1h or 24 h) left ventricles (LV) of mouse heart were separated by SDS-PAGE. Immunoblotting was performed by anti-SUMO1 (**A**) and anti-SUMO2 antibodies (**B**). (**C**,**D**) SUMO protease activities in Sham, Isch, I/R1 and I/R24 heart LV homogenates in SEM buffer was determined by measuring fluorescence signals emitted from liberated AMC substrate (SUMO1-AMC (**C**) vs. SUMO2-AMC (**D**)) over time. RFU: relative fluorescence unit. **E**–**F** SUMO protease activities in Sham, Isch, I/R1 and I/R24 LV heart lysates in SEM buffer was determined by preincubating the lysates for 15 min at 25 °C with either HA-SUMO1-VS (**E**) or HA-SUMO2-VS (**F**) probes followed by separation of the homogenates by SDS-PAGE. Western blotting was performed by adding anti-HA antibody. HA signal was quantified and normalized to vinculin. (**G**) The HA-SUMO2-VS blot (F) was probed with anti-SENP3 antibody. As for negative control, homogenates were treated with NEM (10 mM) to inhibit cysteine protease activity. (**H**) Whole homogenates (1% SDS buffer) from left ventricles (LV) of mouse heart were probed by anti-SENP3 and anti-vinculin antibodies. Vinculin was used as loading control in all cases. Quantification of the respective blots normalized to vinculin are shown. Data are expressed in mean ± SEM, asterisks show significant differences compared to Sham (*n* = 3, * *p* < 0.05, ** *p* < 0.01).

To first investigate how ischemia and reperfusion influences the overall SUMOylation pattern of proteins in mouse heart, we prepared cell homogenates from the left ventricles (LV) of hearts from sham operated mice or mice exposed to ischemia (30 min) or ischemia/reperfusion injury (I/R, 1 h and 24 h). Proper conditions of ischemia and reperfusion were controlled by cardiac troponin I (cTnI) measurement, a sensitive marker of cardiac injury and infarct size [21,22], with the highest cTnI levels in mouse serum after 1 h reperfusion (Figure S1A). Proteins were separated by SDS-PAGE and the SUMO pattern was analyzed by anti-SUMO immunoblotting (Figure 1A,B and Figure S1B,C). Although the most prominent SUMO1 target protein RanGAP1 showed slightly increased SUMOylation after reperfusion (Figure 1A), high molecular weight (HMW) SUMO1 conjugates altogether exhibit a significant decrease after 24 h reperfusion (Figure 1A and Figure S1B). Changes in the SUMO2-conjugated proteins were more dynamic. Following a transient decrease in ischemia, SUMO2 HMW conjugate levels rapidly recovered upon reperfusion (Figure 1B and Figure S1C). Lower molecular weight SUMO conjugates were barely detected on gradient gels (Figure S1B,C). Because SUMO homeostasis is tightly controlled by deSUMOylating enzymes of the SENP family [23], we wanted to investigate whether the amount or activity of SENPs is changed in response to cardiac ischemia and reperfusion. To monitor overall SUMO deconjugase activity in extracts from murine hearts we performed a fluorescence-based activity assay using SUMO1- or SUMO2-AMC probes as substrates ([24]; Figure 1C,D). Upon induction of ischemia and reperfusion, we observed significantly decreased SUMO1- (Figure 1C) - and SUMO2- specific (Figure 1D) deconjugase activities compared to control. Whereas the SUMO1-specific protease activities returned almost to normal (Figure 1C), the SUMO2-specific protease activity did not recover even after 24 h reperfusion (Figure 1D). To determine the activity of distinct SENP family members, we used hemagglutinin (HA)-tagged SUMO-vinylsulfone (VS) derivatives, which function as active site-directed probes for SENPs through irreversible covalent modification of their catalytic cysteine residue [24,25]. When added to a cell extract, active SENPs are labeled by HA-SUMO-VS and can be detected by anti-HA antibody as distinct bands (Figure S1D,E and Figure 1E,F). Addition of the alkylating agent NEM to the cell homogenate blocks the activity of SENPs thus serving as a negative control as visualized by the absence of anti-HA reactive bands in the presence of NEM (Figure S1D,E, first lane). In cell lysates from control mice, major HA-SUMO1-VS-adducts were detected in a molecular weight range between 75 and 100 kD likely correspond to SENP1, SENP2 (Figure S1D), while those between 130 and 180 kD can be attributed to SENP7 and SENP6 (Figure S1D). In case of HA-SUMO2-VS, a prominent protein band was identified at about 95 kD (Figure S1E). By using anti-SENP3 antibody for detection we could confirm that this HA-SUMO2-VS bound species primarily corresponds to SENP3 (Figure 1G and Figure S1F), which functions as the major SUMO deconjugase in the mouse heart. Although the active HA-SUMO2-VS adduct of SENP3 at 95 kD significantly decreased during ischemia and reperfusion, no corresponding increase in the inactive SENP3 form at 75 kD was observed (Figure 1G), suggesting that primarily the protein amount of SENP3 is regulated. Indeed, when total cell lysates of left ventricles were immunoblotted and probed by SENP3 antibody decreased protein amount was detected in I/R injury (Figure 1H).

Altogether, we observe dynamic changes in the overall cellular SUMOylation pattern correlating with decreased SUMO specific isopeptidase (SENPs) activities especially with those of SENP3, during cardiac I/R injury.

### 2.2. Identification of SUMO Targets in Murine Heart

**Figure 2:**
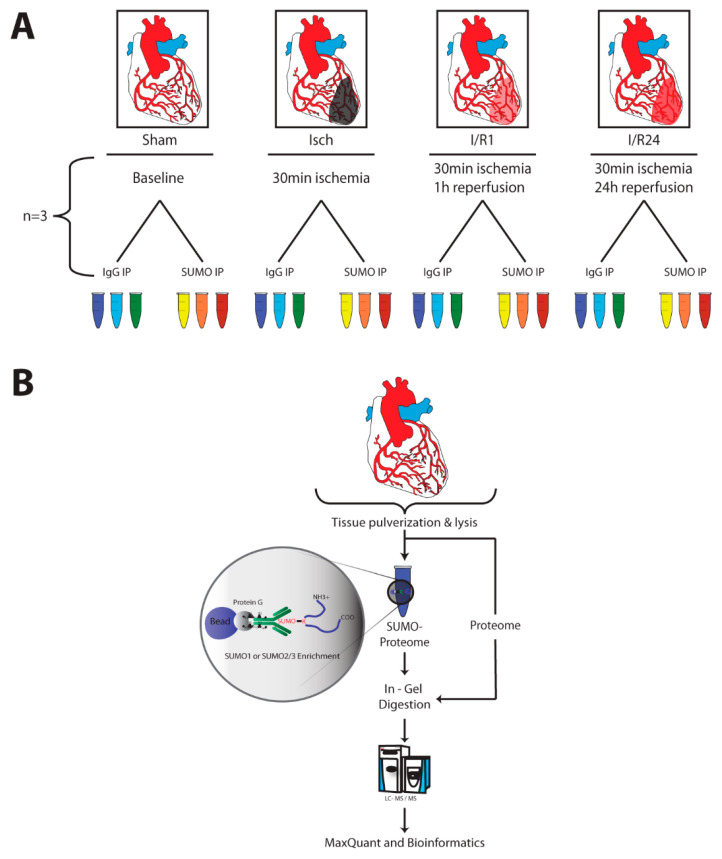
Experimental Setup and workflow of SUMO immunoprecipitation and mass spectrometry. (**A**) Experimental Setup. Mice underwent cardiac surgery in order to ligate left anterior descending artery for 30 min (Isch) and re-open it again for 1 h (I/R1) or 24 h (I/R24) reperfusion. Four mouse hearts for each condition (Sham, Isch, I/R1 or I/R24) were pooled as one biological replicate. The same replicate was used for both SUMO and IgG Control IP. All experiments were performed in triplicates. (**B**) Experimental Workflow of SUMO immunoprecipitation and mass spectrometry. Mouse hearts were snap frozen and pulverized in liquid nitrogen. Powder was lysed in 1% SDS buffer and homogenized in a ball mill. IPs were performed using in-house produced SUMO1-, SUMO2/3- or IgG antibody coupled beads. An aliquot of IP input was used for proteome analysis. IP Eluates representing SUMO1 or SUMO2/3 proteome were separated by SDS-PAGE and subjected to in-gel digestion protocol using trypsin. Peptides were analyzed by a streamlined in-house LC-MS/MS Bioinformatics pipeline.

**Figure 3:**
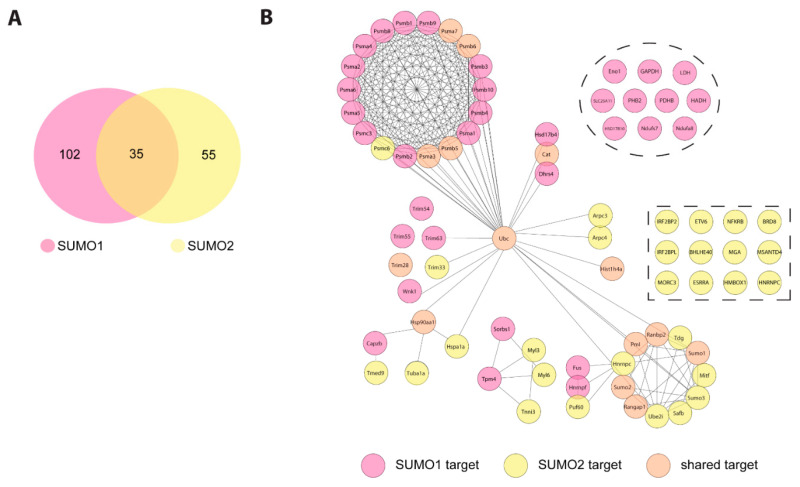
SUMO1 and SUMO2 target protein network in cardiac I/R injury. (**A**) Venn diagram depicting the number of exclusive and overlapping SUMO1 and SUMO2/3 target proteins after imputation of missing values in cardiac I/R injury. (**B**) STRING network of SUMO1 and SUMO2/3 target proteins in cardiac I/R injury. Identified target proteins of both SUMO1 and SUMO2/3 IP were analyzed regarding functional network clustering using STRING database. For STRING analysis, parameters were set at highest confidence level and used MCL clustering with inflation of 3. Proteins are coloured according to their identification in either SUMO1- or SUMO2/3- or both IP’s.

To identify SUMO targets in the mouse heart and to follow the dynamics of these targets during cardiac I/R injury we performed large scale anti-SUMO1 and anti-SUMO2 immunoprecipitations from mouse hearts under different experimental conditions (Figure 2A). To enrich for SUMO conjugates, we followed the protocol described by Barysch et al. [26]. After in-gel digestion, tryptic peptides were analyzed by liquid chromatography (LC) MS/MS, and data from three replicates were quantified by the MaxQuant label-free quantification (LFQ) algorithm (Figure 2B). Among the 400 to 600 identified proteins in each experimental condition (Table S1) we classified proteins as high-confidence SUMO target proteins, if they were at least 1.5-fold (*p* value < 0.05) enriched in SUMO immunoprecipitates versus IgG controls or were exclusively found in SUMO immunoprecipitates but not in IgG ones, in at least two out of three replicates. Applying these criteria we initially identified 73 to 115 SUMO1 and 39 to 53 SUMO2 candidate targets in each experimental condition (Table S1, Figures S2 and S3). In addition to the baits SUMO1 and SUMO2, other well characterized SUMO targets, such as RanBP2, RanGAP1, Ubc9 or PML were highly enriched in anti-SUMO1 or anti-SUMO2 IPs vs. control IPs thus validating our experimental approach (Figures S2 and S3). To allow for relative quantification of those proteins, which were exclusively present in SUMO IPs but not IgG control, the MaxQuant imputation algorithm was applied. 102 proteins were significantly enriched in SUMO1 IP, whereas 55 proteins were identified as candidate SUMO2 targets (Figure 3A, Table S1). We recognized an overlap of 35 shared targets between SUMO1 and SUMO2 immunoprecipitated proteins (Figure 3A). To map functional and physical relationship of these candidate SUMO targets, we analyzed their interconnections using the STRING database (Figure 3B, Table S2). The analysis reveals several functionally distinct clusters of SUMO targets. The largest subgroup of proteins enriched in SUMO1 IPs are connected to components of the SUMO machinery and ubiquitin (Ub) proteasome system. For example, the SUMO E2 Ubc9 (alias Ube2i) as well as SUMO and Ub ligases (TRIM28, PML, RanBP2, Trim 63, 55, and 54 (alias MURFs 1-3)), or the ubiquitin-selective AAA ATPase p97/VCP are identified as bona fide SUMO1 targets (Figure 3B and Figure S2). The modifiers Ub and SUMO2 were also found in SUMO1 immunoprecipitates (IPs) (Figure 3B and Figure S2) indicating either co-modification of proteins with Ub and SUMO or presence of hybrid SUMO1-SUMO2/3 and SUMO-Ub chains. SUMO-Ub hybrid chains are typically generated in response to proteotoxic stress by the StUBL (SUMO-targeted Ub ligase) pathway and are involved in proteasomal disposal of misfolded proteins [27,28]. Intriguingly, multiple subunits of the 20S proteasome core particle are also among the predominant SUMO1 targets (Figure 3B and Figure S2). We identified 17 subunits of the 20S core proteasome as candidate SUMO1 targets (Figure 3B and Figure S2, Table S2). In addition, several metabolic enzymes involved in both glycolytic (Eno1, GAPDH, LDH) and oxidative metabolic pathways (Slc25A11, PDHB, HADH, HSD17B10, Ndufs7 and Ndufa8), emerged as S1 target proteins in the heart (Figure 3B and Figure S2). In SUMO2/3 IP from mouse hearts, we also detected well-known SUMO2 targets (SUMO1, Ube2i (Ubc9), PML, RanGAP1 etc.) (Figure 3B and Figure S3, Table S2). Moreover, we again uncovered ubiquitin and proteasomal subunits (Psma3, Psma7, Psmb5, Psmb6, Psmc6) as most significant cardiac SUMO2 targets (Figure 3B and Figure S3). Additionally, regulators of gene expression and transcription factors are among the most prevalent SUMO2 targets in the heart (Hist1h4a, Top2b, Tdg, Smchd1, Prox1, Brd8, Etv6, BHLH40, Irf2bpl, Irf2bp2, Esrra, Mitf, Mga, Trim24, Safb, SAP130), RNA binding proteins and splicing regulators (Hnrnpc, Sart1, Rbm25), heat shock proteins (Hspa1, hsp90aa1, hsp90b1), as well as special proteins playing important roles in heart function e.g., involved in the regulation of cardiac Ca^2+^ homeostasis or muscle contraction (Tnni3, Myl3, Myl6, Ahnak, Ahnak2) (Figure 3B and Figure S3, Table S2).

### 2.3. Dynamic Changes of SUMO Target Proteins During Cardiac I/R Injury

**Figure 4:**
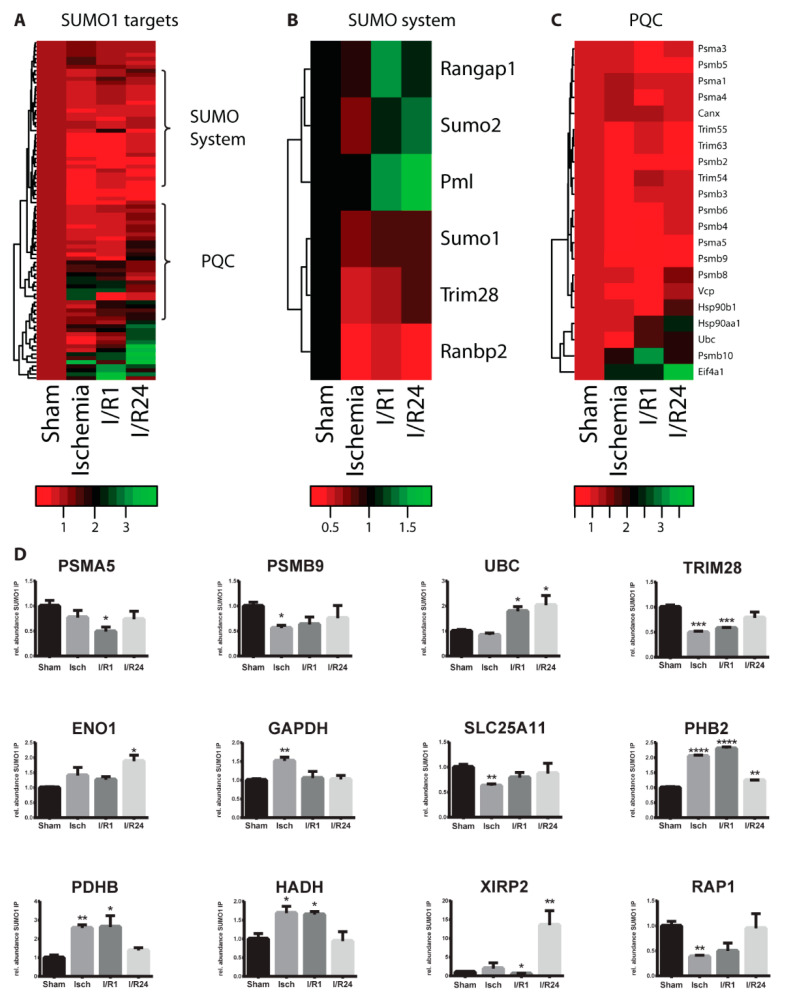
Cardiac I/R injury leads to dynamic changes in SUMO1 proteome. (**A**) Alteration of SUMO1 target proteins upon cardiac ischemia/reperfusion injury (I/R) in the mouse heart. Heat map displays relative changes in SUMO1 targets during I/R when compared to sham control. SUMO1 targets were defined after imputation as follows, difference SUMO IP versus IgG IP log_2_ ratio >0.58 (difference is higher than 1.5×) and -log 10 *p* value > 1.3 (*p* < 0.05). (**B**) Heatmap of a sub-group of SUMO1 target proteins belonging to the SUMO machinery in the mouse heart upon I/R injury. (**C**) Heatmaps of a sub-group of SUMO1 target proteins representing the protein quality control (PQC) machinery in the mouse heart upon I/R injury. (**D**) Bar diagrams of significantly changed SUMO1 targets, when experimental conditions (Isch, I/R1, I/R24) are compared to Sham. Data are expressed in mean ± SEM, asterisks show significant differences compared to Sham (*n* = 3, * *p* < 0.05, ** *p* < 0.01, *** *p* < 0.001, **** *p* < 0.0001).

**Figure 5:**
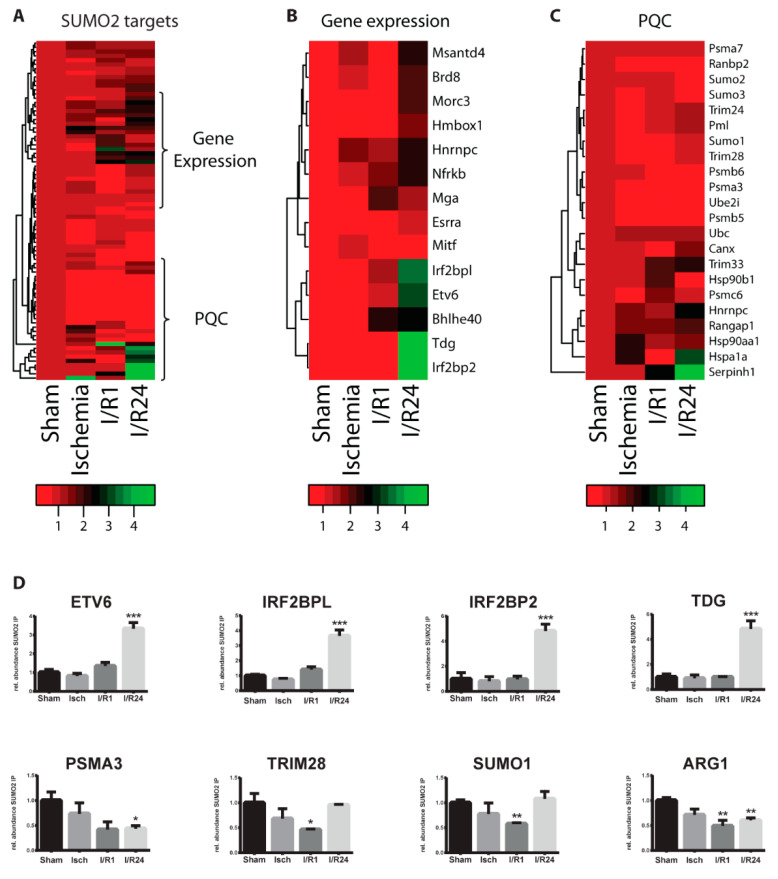
Cardiac I/R injury leads to dynamic changes in SUMO2 proteome. (**A**) Alteration of SUMO2/3 target proteins upon cardiac I/R injury in the mouse heart. The heat map displays relative changes in SUMO2/3 targets during I/R when compared to sham control. SUMO2/3 targets were defined after imputation as follows, difference SUMO2/3 IP versus IgG IP log_2_ ratio > 0.58 (difference is higher than 1.5×) and -log 10 *p* value > 1.3 (*p* < 0.05). (**B**) Heatmap of a sub-group of SUMO2/3 target proteins playing a role in the regulation of gene expression in the mouse heart upon I/R injury. (**C**) Heatmap of a sub-group of SUMO2/3 target proteins belonging to the protein quality control machinery (UPS, SUMO system, chaperones) in the mouse heart upon I/R injury. (**D**) Bar diagrams of significantly changed SUMO2/3 targets, when experimental conditions (Isch, I/R1, I/R24) are compared to Sham. Data are expressed in mean ± SEM, asterisks show significant differences compared to Sham (*n* = 3, * *p* < 0.05, ** *p* < 0.01, *** *p* < 0.001).

To visualize the SUMOylation dynamics of SUMO target proteins in the course of cardiac ischemia and reperfusion we prepared heat maps for functionally-related protein groups (Figure 4A–C and Figure 5A–C). Most SUMO target proteins exhibited only slight alterations throughout ischemia and reperfusion (Figure 4A–C and Figure 5A–C). For example, the three muscle-specific E3 ubiquitin ligases TRIM63, TRIM55, and TRIM54 (or MURF1-3) which were among the highly significant SUMO1 targets in mouse heart, did not undergo dramatic changes in response to ischemia and reperfusion (Figure 4C). However, a sub-group of proteins showed significant changes in their SUMOylation dynamics (Figure 4D, Figure S4, Figure 5D and Figure S5). Total proteome analysis by mass spectrometry demonstrates that most of the significantly regulated targets did not exhibit changes in their steady state levels (not shown), indicating that indeed SUMOylation and not only the protein amount of these candidate targets was altered. Altogether, 24 candidate SUMO1 target proteins underwent dynamic changes in SUMO conjugation during cardiac I/R injury (Figure 4D and Figure S4). 15 proteins changed significantly their SUMOylation pattern during ischemia, while another 13 and 12 proteins showed altered SUMOylation following 1 h and 24 h reperfusion, respectively (Table S3). The nuclear import-export regulator RanGAP1 and PML, which is the core component of PML nuclear bodies, represent the most abundant cellular SUMO targets. Interestingly, both showed increased SUMO1 conjugation during reperfusion when compared to sham control group (Figure 4B). In case of RanGAP1, increased SUMO1ylation in reperfusion was confirmed by SUMO1 Western blot from total protein lysates of left ventricles (Figure 1A). Notably, PML exhibits a continuous increase in SUMO1 conjugation up to 24 h after reperfusion. SENP3 was previously identified as SUMO deconjugase for PML [29] possibly indicating that reduced SENP3 activity under cardiac I/R (Figure 1F,G) may contribute to the enhanced SUMOylation of PML. The functional significance of increased PML SUMOylation in cardiac I/R is not clear yet. However, considering that PML prevents apoptotic cell death after hypoxia–ischemia in murine brain [30,31] it is attractive to speculate that SUMOylation contributes to these processes in the heart. It is worth noting that both ubiquitin and SUMO2 were enriched in SUMO1 IPs upon reperfusion (Figure 4B–D). This finding is consistent with the stress-induced co-modification of substrates with SUMO1/2 and ubiquitin or the formation of hybrid SUMO1/2-Ub chains under these conditions. Intriguingly, SUMO-targeted ubiquitination is considered to cope with proteotoxic stress by targeting misfolded proteins for proteasomal degradation at or near PML nuclear bodies [32]. Strikingly, several subunits of the 20S proteasome core particle as well as the Ub-shuttling factor p97/VCP exhibit a transient decrease in SUMO1 conjugation in ischemia and 1h reperfusion before recovery towards normal levels upon 24h reperfusion (Figure 4C,D) suggesting that the UPS system is a major target for SUMO controlled adaptations to I/R injury. In addition to the UPS system, transcription factors and transcriptional regulators are enriched among altered SUMO1 targets. These include H4 Histone (Hist1H4A), the transcription factor Prox1 and the gene expression regulators SMCHD1, Trim28 and Isoc2a (Figure 4D and Figure S4). Moreover, several metabolic enzymes which we identified as SUMO1 targets in the heart (Figure 3B), are significantly regulated upon ischemia and reperfusion (Figure 4D): the glycolytic enzymes enolase (ENO1) and GAPDH, the mitochondrial malate-alpha-ketoglutarate transporter (SLC25A11), prohibitin 2 (Phb2), a chaperone to stabilize mitochondrial respiratory chain enzymes, as well as oxidative enzymes, like the E1 enzyme of the PDH complex (PDHB), or the mitochondrial beta-oxidation enzyme, 3-hydroxyacyl-CoA dehydrogenase (HADH). Previous reports have shown that the activity of key glycolytic enzymes e.g., GAPDH are facilitated by increased SUMO1ylation upon hypoxia [33] which is consistent with our finding on enhanced SUMOylation of GAPDH during cardiac ischemia (Figure 4D). Our data indicate that alterations in SUMO dynamics impact on metabolic reprogramming in response to ischemia and reperfusion. However, future work needs to unravel the functional relevance. To note, VDAC1 and 2, the voltage sensitive anion channel proteins regulating also apoptosis [34,35], XIRP2, a muscle-specific, actin binding Xin gene family member and a protective factor localized in the intercalated disc critical for proper cardiac conduction [36], and RAP1, a small GTPase activating protein and a novel modulator of hypoxia-induced apoptosis with an important role in cardiac I/R injury [37] are all among the regulated SUMO1 targets in the heart (Figure 4D and Figure S4).

Similar to SUMO1, cardiac SUMO2 target proteins underwent a dynamic adaptation process in cardiac I/R (Figure 5A). While only a single protein was differentially SUMOylated in ischemia (Clic1), 8 and 11 target proteins underwent significant changes after 1 h vs. 24 h reperfusion, respectively (Table S3). Functional classification of these regulated SUMO2 targets reveals an enrichment of transcriptional regulators, including Etv6, BHLHE40, IRF2BPl and IRF2BP2 (Figure 3B and Figure 5B,D). We have recently identified BHLHE40 as a prominent SUMO target under hypoxia and demonstrated increased repressive potential of BHLHE40 upon SUMOylation [23]. Similarly, the Melchior group reported that IRF2BP1/2 act as SUMO-dependent repressors on immediate early gene transcription [38]. IRF2BPl and mainly, IRF2BP2, a transcription factor binding on NFAT promotors, was previously described to act as a co-activator of vascular endothelial growth factor-A (VEGFA) in cardiac and skeletal muscle [39]. These data indicate that SUMOylation of hypoxia or angiogenesis-related transcriptional regulators contributes to cardiac adaptation to I/R injury. Additionally, we also identified decreased SUMO2ylation of the 20S proteasome core particle subunits (e.g., Psmb5, Psmb6, Psma3) and of SUMO1 and Trim28 in reperfusion (Figure 5C,D). In contrast to SUMO1 proteome, metabolic enzymes were not typically modified by SUMO2 in mouse heart, however, the extrahepatic Arginase 1 (ARG1), competing for Arg with NO synthase and known to be induced very fast in cardiac ischemia [40] showed decreased SUMO2ylation. Interestingly, EIF6, a ribosome biogenesis and translation initiation factor, and PTRF (or Cavin-1), whose deficiency leads to cardiac hypertrophy [41] and can also promote ribosomal transcriptional activity [42], showed altered SUMO2ylation in early reperfusion (Figure S5). Similarly, Ahnak2, a regulator of cardiac L-type Ca^2+^ channels [43] and the mitochondrial chloride channel Clic1 [44] exhibited significantly altered SUMO2ylation in response to cardiac ischemia and reperfusion as well (Figure S5).

## 3. Conclusions

In summary, we provide here the first unbiased proteomic analysis of SUMO targets in mouse heart under conditions of ischemia-reperfusion injury. Although our data are mainly of descriptive nature, the datasets can serve as a resource for future functional studies. Notably, SUMO proteomics on endogenous proteins is still a challenging task, in particular when working with whole organs and tissues. Lumpkin et al. [45] were the first to analyze SUMOylation in the heart and although 30 unique SUMO targets were described, they were not able to distinguish between SUMO1 and SUMO2 modification. Recently, Hendriks at al. [46] used another approach and across all mouse organs analyzed, wild type heart represented the lowest number (about 200) of SUMO2 sites. Although with our method [26] we could not map the exact sites of SUMOylation, and we were not able to determine the exact cellular origin of the detected proteins, we identify here about 100 potential SUMO1, 50 SUMO2 and 35 shared targets in a highly relevant pathological condition of the heart. Some of the significantly regulated SUMO targets presented here, have been also found in wild type heart (e.g., GAPDH, Trim28, Prox1, Ubc or Etv6; [45,46]) (Table S4), some others were detected in other tissues or cell lines (e.g., Eno1, Arg1, Irf2bpl) validating our experimental approach. However, we could also identify potential SUMO targets which were not described yet (e.g., Psmb9, Hadh, Phb2 or Ptrf) (Table S4). Although our dataset likely covers only a subset of cardiac SUMO targets, some important conclusions can be drawn from these data. First, dynamic changes in the overall SUMOylation pattern correlated with decreased SUMO specific isopeptidase (SENPs) activities especially with those of SENP3. So far, only SENP1 and SENP3 were connected to the pathomechanism of cardiac I/R injury [17,18,19,20,47,48]. The literature is very controversial about the role of SENP3 ascribing both protective and detrimental effects to SENP3 on cardiac function [17,18,19,20]. In our experiments, decreased SENP3 activities correlated with enhanced SUMOylation of PML, the main organizer of nuclear bodies which are considered to cope with proteotoxic stress by targeting misfolded proteins for proteasomal degradation. Since we detected Ub in SUMO proteome and several subunits of the 20S proteasome core particle, it is very likely that SUMOylation plays a central role in proteotoxic stress adaptation by regulating PML, the StUBL pathway and the ubiquitin proteasome system in cardiac I/R. Another important finding is, that several metabolic enzymes and hypoxia- or angiogenesis related transcription factors show regulated SUMOylation pattern either by SUMO1 or SUMO2, likely coordinating metabolic reprogramming of the ischemic heart posttranslationally. However, since we analyzed young mice in our experiments, one has to be cautious by transferring the results for human coronary disease. Further studies are certainly required to delineate functional significance of the regulated SUMO targets in ischemic adaptation of the heart.

## 4. Materials and Methods

### 4.1. Animal Model for Cardiac Ischemia and Reperfusion

Experiments involving animals were conducted at the Max Planck Institute for Heart and Lung Research in Bad Nauheim, Germany accordance with institutional guidelines and laws, following protocols approved by local animal ethics committees and authorities (Regierungspraesidium Darmstadt, V 54–19 c 20/15 - FU-1156). 12–14-weeks-old C57BL/6J male mice were purchased for Charles River (Germany) and kept in temperature- and humidity-controlled animal facility at 12 h of light-to-dark cycles with access to water and food ad libitum. For anaesthesia induction, mice were exposed to 4.5 vol% isoflurane in ambient air for approximately 2 min in an air-tight box. Anaesthesia was maintained after intra-tracheal intubation and ventilation applying 1.5 vol% isoflurane using the rodent MiniVent ventilator (Harvard Apparatus, HSE) adjusted to 220 hubs per minutes with a tidal volume of 300 μL. During the procedure, animals were kept in a supine position on a heat-controlled plate at 37 °C. A left anterolateral thoracotomy was performed between the second and third rib to visualize the mouse heart and the LAD. The LAD was ligated in a proximal position using a 7-0 prolene suture. Pale discoloration of ventricular tissue demarcated the region of ischemia. In Sham group, thoracotomy was similarly done but no ligation occurred. After ligation the open wound was covered using cheese cloth soaked with 0.9% NaCl solution. After 30 min of ischemia, the mice were assigned to either ischemia (Isch) or ischemia/reperfusion groups (I/R). In Isch group, ligated hearts were in situ perfused with PBS and immediately removed from the mice. In I/R groups, ligation was opened after 30 min ischemia and cardiac reperfusion confirmed by visual control. The wound was closed using absorbable, synthetic 5-0 vicryl (polyglactin 910) sutures. Weaning from ventilation was ended when spontaneous respiration became evident. After 1 h or 24 h reperfusion time (I/R1 and I/R24 group, respectively), mice were anesthetized by ketamin/xylazine, hearts were perfused with PBS and removed from the animals. In all 4 groups (Sham, Isch, I/R1, I/R24), left ventricle (LV) was separated from septum and right ventricle, weighed, cut in small pieces, immediately frozen in liquid nitrogen and kept in −80 °C until use. Blood for cTnI ELISA measurements was collected from all mouse hearts before PBS perfusion, and serum was kept at −80 °C until use.

### 4.2. Cardiac Troponin-I ELISA

A commercially available kit was used to quantify cardiac isoform of Troponin I from serum of experimental animals as measure of I/R injury of the heart (Ultra-sensitive mouse cardiac Troponin-I ELISA, Life Diagnostics). The assay was performed according to manufacturer’s instructions.

### 4.3. Tissue Homogenization

Mouse LVs were homogenized in 10× volume 1% SDS buffer pH = 7.4 with protease inhibitors (Complete, Roche) and 10 mM NEM for Western blotting and MS experiments [26]. For Western blotting, Potter homogenizer (Wheaton, 1 mL) was used and homogenates were centrifuged at 18,000 *g* for 15 min. The protein concentration of the supernatant was measured (DC assay, Bio-Rad), mixed with Laemmli sample buffer and heated for 5 min at 97 °C.

For SUMO protease activity measurements, LVs were homogenized in 10× volume SEM buffer (0.25 M sucrose, 20 mM MOPS-KOH (pH 7.4), 1 mM EDTA-NaOH pH 8, 1 mM DTT); [24]) with protease inhibitors (Complete, Roche) and without NEM in Potter homogenizer at 4 °C. The homogenates were centrifuged at 18,000 *g* for 10 min at 4 °C, supernatant was taken and protein concentration was measured (DC assay, Bio-Rad).

### 4.4. SUMO1/2-AMC Cleavage Assays for SUMO Protease Activity Measurements

AMC assays were performed as described [24]. Shortly, freshly homogenized LVs in SEM buffer (30 μg protein for SUMO1-AMC and 15 μg protein for SUMO2-AMC) were pipetted into a 384-well plate on ice. For negative control, 10 mM NEM was added to the lysate so that SUMO protease activity was blocked. SEM buffer (without lysate) was used as blank. An assay master-mix was then added to the homogenates; for each reaction 500 nM SUMO1-AMC or SUMO2-AMC substrate (Boston Biochem) was adjusted with activity assay buffer (50 mM Tris-HCl (pH 7.5), 0.1 mg/mL BSA, 10 mM DTT) to a total volume of 50μL including volume of lysate. The plate was quickly spinned down to collect liquids and the measurement was immediately started on a Plate reader (BioTek) at λ_Ex_ = 380 nm, λ_Em_ = 460 nm. At least three replicates for each condition were measured.

### 4.5. SUMO1/2-VS Adduct Formation Assays for SUMO Protease Activity Measurements

SUMO1/2-VS assays were done as described [24]. Shortly, 200 μg of heart LV lysates prepared in SEM buffer were incubated with 100 ng of either HA-SUMO1-VS or HA-SUMO2-VS probes (Boston Biochem) for 15 min at 25 °C. The reaction was stopped by addition of Laemmli sample buffer (5 min, 97 °C).

### 4.6. SDS-PAGE and Western Blot Analysis

To analyze the global SUMOylation status of LVs during ischemia and reperfusion, 40 μg of 1% SDS lysates of LVs was used containing also NEM (10 mM) to block the activity of SUMO proteases. Standard SDS-PAGE under reducing conditions was carried out followed by wet transfer onto 0.2 μm nitrocellulose membrane. SUMO-protein conjugates were analyzed by applying SUMO1 (1:20–1:40 diluted supernatant of 21C7 mouse hybridoma cells) or SUMO2 antibodies (1:2000 mouse, M114-3, MBL) in 5% milk powder in PBS-T. The protein amount of SENP3 (1:5000, rabbit, D20A10, Cell Signaling) was also measured from the same lysates.

To follow the SUMO protease activities, 40 μg LV SEM lysates incubated with either HA-SUMO1-VS or HA-SUMO2-VS probes were loaded on the gel followed by the same procedure as for SDS lysates. The HA-SUMO1/2-VS bound adducts of SUMO proteases were then visualized by anti-HA antibody (1:2000, mouse, MMS-101R, Covance/Biolegend) or by anti-SENP3. In all Western blots vinculin was used as loading control (1:80,000, V9131, Sigma Aldrich).

### 4.7. SUMO Immunoprecipitation (IP)

SUMO1-specific 21C7 hybridoma cells and SUMO2/3 specific 8A2 hybridoma clone A11 (kindly received from Frauke Melchior) were incubated at 37 °C and 5% CO_2_ using RPMI medium supplemented with 10% FBS, 1% Penicillin/Streptomycin and 0.1% gentamycin. 25 Million hybridoma cells were transferred to hybridoma serum-free-medium and cultured for 7 to 10 days in a CELLine CL 1000 incubator according to manufacturer’s instructions. Cells were collected and centrifuged at 100 *g* room temperature for 5 min. Antibody containing supernatant was removed and centrifuged again at 20,000 *g* for 20 min at 4 °C to remove cell debris. Antibody concentration was estimated by comparing different amounts of supernatant with different amounts of normal mouse IgG (known concentration) on a Coomassie-stained SDS-Gel. Antibodies were dialyzed at 4 °C overnight against a 30-fold access of sodium phosphate buffer (20 mM NaH2PO4/Na2HPO4 pH 7.0) using a dialysis membrane Spectra/Por4 (MWCO 12–14,000 Da). Antibody coupling to beads and SUMO1 or SUMO2/3 immunoprecipitation were performed based on a published protocol [26]. Shortly, mouse hearts were pulverized in liquid nitrogen. Four mouse heart LVs (about 180–200 mg heart tissue) were pooled for each biological replicate for tissue homogenization and three replicates were used for each experimental condition (12 hearts/condition (Sham, Isch, I/R1, I/R24)). The homogenization was done in 2 mL Eppendorf tubes in Retsch mixer mill (MM400) for 3 min at maximal speed (30 rpm). After centrifugation of the Eppendorf tubes at 18,000 g for 15 min, supernatants were taken and protein concentration was measured as described. A small aliquot of each replicate input was taken for proteome analysis, the rest was further processed for SUMO proteome measurements. Half of each replicate (representing about 100 mg original heart tissue and 10 mg isolated protein) were taken for SUMO IP and the other half for IgG control IP. SUMO1 and SUMO2/3 IPs were performed from different experimental hearts. The efficiency of SUMO1 and SUMO2/3 IPs were controlled by Western blot.

### 4.8. Sample Preparation, Liquid Chromatography and Mass Spectrometry (MS)

Input proteome samples (6 replicates) as well as the immunoprecipitated SUMO proteome replicates (triplicates for SUMO1/IgG and triplicates for SUMO2/IgG) were separated by gradient gels (4–20%, BioRad or 4–12% Bis-Tris gels, Invitrogen) followed by protein staining (Instant Blue, Expedeon) as described recently [49]. The individual gel lanes were cut into equal, small pieces and in-gel digestion with sequencing-grade trypsin was performed as described [50]. Finally, extracted peptides were collected, concentrated, and desalted using the Stop and Go Extraction (STAGE) technique [51].

Peptides from individual gel bands were analyzed on a Q Exactive HF coupled to an easy nLC 1200 (ThermoFisher Scientific) using a 20 cm long, 75µm ID fused-silica column packed in house with 1.9 µm C18 particles (Reprosil pur, Dr. Maisch), and kept at 50 °C using an integrated column oven (Sonation). Peptides were eluted by a non-linear gradient from 8–30% acetonitrile over 24 min (IPs) or 4–30% acetonitrile over 58 min (proteomes) and directly sprayed into the mass-spectrometer equipped with a nanoFlex ion source (ThermoFisher Scientific). The mass spectrometer was operated in a data-dependent mode. Full-scan MS spectra of IPs/proteomic samples were acquired covering a mass range of 300–1650 *m*/*z* using 3E6 as AGC target with a resolution of 60.000 at 200 *m*/*z* and a maximum injection time of 20 ms. The 15 most intense ions were fragmented by Higher-energy collisional dissociation (HCD) applying a normalized collision energy of 27. Resolution for MS/MS spectra was set to 30.000/15.000 at 200 *m*/*z*, AGC target to 1E5, maximal injection time to 64 ms/30 ms.

All acquired raw files of mass spectra were analyzed using MaxQuant Software (version 1.5.8.3) [52,53] and the implemented Andromeda database search engine [54]. Fragmentation spectra were correlated with the Uniprot mouse Reference Proteome database including isoforms (downloaded September 2017, 60,163 sequences). To perform searches, tryptic digestion and default settings for mass tolerances of MS and MS/MS spectra were applied. False discovery rate (FDR) was set to 1%, minimal LFQ ratio count was set to 2 and FastLFQ option was enabled for relative label-free quantification of proteins using the in-build MaxLFQ algorithm [55]. For both IP/MS screens the match between run feature was used. For the endogenous SUMO IPs, the minimal razor + unique peptides for identification were set to 1 and unique + razor peptides were allowed for protein quantification. Visual representation of data in volcano plots was done using the R Studio software (version 1.1.463).

### 4.9. Quantification and Statistical Analysis

Proteome experiments were performed in 6 replicates and SUMO IPs in triplicates. MS data analysis and statistics were done with the Perseus software (version 1.5.8.5). First, contaminants and reverse entries, as well as proteins only identified by a modified peptide were removed. The log2 value of all LFQ intensities was calculated. Using the e4 Cell Reports 29, 480–494.e1–e5, October 8, 2019 histogram analysis function of the software, normal distribution of the LFQ values was visually checked. Good correlation of the experimental replicates was assured by multiscatter plot analysis. Samples were then grouped into triplicates and a Student’s t test was performed with a randomization of 500 and a s0 factor of 0.1. To overcome the missing value problem, imputation of the SUMO1 and SUMO2 IP datasets was done in Perseus using default settings. Prior to any replacement of missing values by normal distribution, the matrix was filtered for at least three valid values in total and rows not matching these criteria were discarded. LFQ values and results of the t test after imputation were combined with the matrix prior to imputation. Then the datasets were exported and used for further analysis in Microsoft Excel. Significant enrichment was defined in Excel based on the *p* value and the Student’s t test difference applying following criteria for the IP dataset: -log10 *p*-value > 1.3 and log2 ratio 0.58 (1.5× enrichment over IgG IP) and for the proteome -log 10 *p* value > 1.3 and log2 ratio R −0.265 < x > 0.265.

For the generation of the STRING networks, the freely available STRING database was used (https://string-db.org (version 10.5)). For network analysis the criteria were set as follows: SUMO target proteins with a log2 ratio > 0.58 over IgG IP were included. For all analyses the parameters were set to highest confidence. Experiments and databases were enabled.

### 4.10. Data Availability

The mass spectrometry proteomics data have been deposited to the ProteomeXchange Consortium [56] via the PRIDE partner repository [57] with the dataset identifier PXD021640.

## Supplementary Materials

The following are available online, Supplementary Figures and Tables (pdf) and Supplementary Table S2 (xlsx).

## References

[1] Murray C.J., Lopez A.D. (1997). Mortality by cause for eight regions of the world: Global Burden of Disease Study. Lancet.

[2] Heusch G. (2020). Myocardial ischaemia–reperfusion injury and cardioprotection in perspective. Nat. Rev. Cardiol..

[3] Chandrashekhar Y., Sen S., Anway R., Shuros A., Anand I. (2004). Long-term caspase inhibition ameliorates apoptosis, reduces myocardial troponin-I cleavage, protects left ventricular function, and attenuates remodeling in rats with myocardial infarction. J. Am. Coll. Cardiol..

[4] Kairouz V., Lipskaia L., Hajjar R.J., Chemaly E.R. (2012). Molecular targets in heart failure gene therapy: Current controversies and translational perspectives. Ann. N. Y. Acad. Sci..

[5] Münzel T., Gori T., Keaney J.F., Maack C., Daiber A. (2015). Pathophysiological role of oxidative stress in systolic and diastolic heart failure and its therapeutic implications. Eur. Hear. J..

[6] Flotho A., Melchior F. (2013). Sumoylation: A Regulatory Protein Modification in Health and Disease. Annu. Rev. Biochem..

[7] Mendler L., Braun T., Müller S. (2016). The Ubiquitin-Like SUMO System and Heart Function. Circ. Res..

[8] Hickey C.M., Wilson N.R., Hochstrasser M. (2012). Function and regulation of SUMO proteases. Nat. Rev. Mol. Cell Biol..

[9] Kunz K., Piller T., Müller S. (2018). SUMO-specific proteases and isopeptidases of the SENP family at a glance. J. Cell Sci..

[10] Nayak A., Müller S. (2014). SUMO-specific proteases/isopeptidases: SENPs and beyond. Genome Biol..

[11] Müller S., Hoege C., Pyrowolakis G., Jentsch S. (2001). Sumo, ubiquitin’s mysterious cousin. Nat. Rev. Mol. Cell Biol..

[12] Ulrich H.D. (2008). The Fast-Growing Business of SUMO Chains. Mol. Cell.

[13] Jansen N.S., Vertegaal A.C. (2020). A Chain of Events: Regulating Target Proteins by SUMO Polymers. Trends Biochem. Sci..

[14] Sriramachandran A.M., Dohmen R.J. (2014). SUMO-targeted ubiquitin ligases. Biochim. et Biophys. Acta (BBA) Bioenerg..

[15] Mukhopadhyay D., Dasso M. (2007). Modification in reverse: The SUMO proteases. Trends Biochem. Sci..

[16] Yang W., Sheng H., Thompson J.W., Zhao S., Wang L., Miao P., Liu X., Moseley M.A., Paschen W. (2014). Small Ubiquitin-Like Modifier 3–Modified Proteome Regulated by Brain Ischemia in Novel Small Ubiquitin-Like Modifier Transgenic Mice. Stroke.

[17] Gao L., Zhao Y., He J., Yan Y., Xu L., Lin N., Ji Q., Tong R., Fu Y., Gao Y. (2018). The desumoylating enzyme sentrin-specific protease 3 contributes to myocardial ischemia reperfusion injury. J. Genet. Genom..

[18] Zhang Y., Zheng L.-M., Wang C.-X., Gu J.-M., Xue S. (2018). SENP3 protects H9C2 cells from apoptosis triggered by H/R via STAT3 pathway. Eur. Rev. Med Pharmacol. Sci..

[19] Bian X., Xu J., Zhao H., Zheng Q., Xiaozhi L., Ma X., Li Y., Du X., Liu X. (2019). Zinc-Induced SUMOylation of Dynamin-Related Protein 1 Protects the Heart against Ischemia-Reperfusion Injury. Oxidative Med. Cell. Longev..

[20] Rawlings N., Lee L., Nakamura Y., Wilkinson K.A., Henley J.M. (2019). Protective role of the deSUMOylating enzyme SENP3 in myocardial ischemia-reperfusion injury. PLoS ONE.

[21] Engle S.K., Jordan W.H., Pritt M.L., Chiang A.Y., Davis M.A., Zimmermann J.L., Rudmann D.G., Heinz-Taheny K.M., Irizarry A.R., Yamamoto Y. (2009). Qualification of Cardiac Troponin I Concentration in Mouse Serum Using Isoproterenol and Implementation in Pharmacology Studies to Accelerate Drug Development. Toxicol. Pathol..

[22] Frobert A., Valentin J., Magnin J.-L., Riedo E., Cook S., Giraud M.-N. (2015). Prognostic Value of Troponin I for Infarct Size to Improve Preclinical Myocardial Infarction Small Animal Models. Front. Physiol..

[23] Kunz K., Wagner K., Mendler L., Hölper S., Dehne N., Muller S.C. (2016). SUMO Signaling by Hypoxic Inactivation of SUMO-Specific Isopeptidases. Cell Rep..

[24] Kunz K., Muller S.C., Mendler L. (2019). Assays of SUMO protease/isopeptidase activity and function in mammalian cells and tissues. Methods Enzym..

[25] Madu I.G., Chen Y. (2012). Assays for Investigating deSUMOylation Enzymes. Curr. Protoc. Mol. Biol..

[26] Barysch S.V., Dittner C., Flotho A., Becker J., Melchior F. (2014). Identification and analysis of endogenous SUMO1 and SUMO2/3 targets in mammalian cells and tissues using monoclonal antibodies. Nat. Protoc..

[27] Gärtner A., Muller S.C. (2014). PML, SUMO, and RNF4: Guardians of nuclear protein quality. Mol. Cell.

[28] Keiten-Schmitz J., Wagner K., Piller T., Kaulich M., Alberti S., Müller S. (2020). The Nuclear SUMO-Targeted Ubiquitin Quality Control Network Regulates the Dynamics of Cytoplasmic Stress Granules. Mol. Cell.

[29] Han Y., Huang C., Sun X., Xiang B., Wang M., Yeh E.T.H., Chen Y., Li H., Shi G., Cang H. (2010). SENP3-mediated De-conjugation of SUMO2/3 from Promyelocytic Leukemia Is Correlated with Accelerated Cell Proliferation under Mild Oxidative Stress. J. Biol. Chem..

[30] Bernardi R., Papa A., Pandolfi P.P. (2008). Regulation of apoptosis by PML and the PML-NBs. Oncogene.

[31] Palibrk V., Suganthan R., Scheffler K., Wang W., Bjørås M., Bøe S.O. (2016). PML regulates neuroprotective innate immunity and neuroblast commitment in a hypoxic–ischemic encephalopathy model. Cell Death Dis..

[32] Lallemand-Breitenbach V., Jeanne M., Benhenda S., Nasr R., Lei M., Peres L., Zhou J., Raught B., De Thé H. (2008). Arsenic degrades PML or PML–RARα through a SUMO-triggered RNF4/ubiquitin-mediated pathway. Nat. Cell Biol..

[33] Agbor T.A., Cheong A., Comerford K.M., Scholz C.C., Bruning U., Clarke A., Cummins E.P., Cagney G., Taylor C.T. (2010). Small Ubiquitin-related Modifier (SUMO)-1 Promotes Glycolysis in Hypoxia. J. Biol. Chem..

[34] Naghdi S., Hajnóczky G. (2016). VDAC2-specific cellular functions and the underlying structure. Biochim. et Biophys. Acta (BBA) Bioenerg..

[35] Shoshan-Barmatz V., Shteinfer-Kuzmine A., Verma A. (2020). VDAC1 at the Intersection of Cell Metabolism, Apoptosis, and Diseases. Biomol..

[36] Huang L., Wu K., Zhang L., Wang Q., Tang S., Wu Q., Jiang P., Lin J.J., Guo J., Wang L. (2018). Critical Roles of Xirp Proteins in Cardiac Conduction and Their Rare Variants Identified in Sudden Unexplained Nocturnal Death Syndrome and Brugada Syndrome in Chinese Han Population. J. Am. Hear. Assoc..

[37] Cai Y., Ying F., Liu H., Ge L., Song E., Wang L., Zhang D., Tang E.H.C., Xia Z., Irwin M.G. (2020). Deletion of Rap1 protects against myocardial ischemia/reperfusion injury through suppressing cell apoptosis via activation of STAT3 signaling. FASEB J..

[38] Barysch  S.V., Stankovic-Valentin N., Karaca S., Doppel J., Achour T.N., Sticht C., Urlaub H., Melchior F. (2019). Transient deSUMOylation of IRF2BP proteins controls early transcription in EGFR signaling. bioRxiv.

[39] Teng A.C.T., Kuraitis D., Deeke S.A., Ahmadi A., Dugan S.G., Cheng B.L.M., Crowson M.G., Burgon P.G., Suuronen E.J., Chen H.-H. (2010). IRF2BP2 is a skeletal and cardiac muscle-enriched ischemia-inducible activator of VEGFA expression. FASEB J..

[40] Schlüter K.-D., Schulz R., Schreckenberg R. (2015). Arginase induction and activation during ischemia and reperfusion and functional consequences for the heart. Front. Physiol..

[41] Taniguchi T., Maruyama N., Ogata T., Kasahara T., Nakanishi N., Miyagawa K., Naito D., Hamaoka T., Nishi M., Matoba S. (2016). PTRF/Cavin-1 Deficiency Causes Cardiac Dysfunction Accompanied by Cardiomyocyte Hypertrophy and Cardiac Fibrosis. PLoS ONE.

[42] Jansa P., Burek C., Sander E.E., Grummt I. (2001). The transcript release factor PTRF augments ribosomal gene transcription by facilitating reinitiation of RNA polymerase I. Nucleic Acids Res..

[43] Haase H. (2007). Ahnak, a new player in β-adrenergic regulation of the cardiac L-type Ca2+ channel. Cardiovasc. Res..

[44] Ponnalagu D., Gururaja-Rao S., Farber J., Xin W., Hussain A.T., Shah K., Tanda S., Berryman M., Edwards J.C., Singh H. (2016). Molecular identity of cardiac mitochondrial chloride intracellular channel proteins. Mitochondrion.

[45] Lumpkin R.J., Gu H., Zhu Y., Leonard M., Ahmad A.S., Clauser K.R., Meyer J.G., Bennett E.J., Komives E.A. (2017). Site-specific identification and quantitation of endogenous SUMO modifications under native conditions. Nat. Commun..

[46] Hendriks I.A., Lyon D., Su D., Skotte N.H., Daniel J.A., Jensen L.J., Nielsen M.L. (2018). Site-specific characterization of endogenous SUMOylation across species and organs. Nat. Commun..

[47] Guo C., Hildick K.L., Luo J., Dearden L.A., Wilkinson K., Henley J.M. (2013). SENP3-mediated deSUMOylation of dynamin-related protein 1 promotes cell death following ischaemia. EMBO J..

[48] Gu J., Fan Y., Liu X., Zhou L., Cheng J., Cai R., Xue S. (2014). SENP1 protects against myocardial ischaemia/reperfusion injury via a HIF1α-dependent pathway. Cardiovasc. Res..

[49] Wagner K., Kunz K., Piller T., Tascher G., Hölper S., Stehmeier P., Keiten-Schmitz J., Schick M., Keller U., Muller S.C. (2019). The SUMO Isopeptidase SENP6 Functions as a Rheostat of Chromatin Residency in Genome Maintenance and Chromosome Dynamics. Cell Rep..

[50] Shevchenko A., Tomas H., Havlis J., Olsen J.V., Mann M.J. (2006). In-gel digestion for mass spectrometric characterization of proteins and proteomes. Nat. Protoc..

[51] Rappsilber J., Ishihama Y., Mann M. (2003). Stop and Go Extraction Tips for Matrix-Assisted Laser Desorption/Ionization, Nanoelectrospray, and LC/MS Sample Pretreatment in Proteomics. Anal. Chem..

[52] Cox J., Mann M. (2008). MaxQuant enables high peptide identification rates, individualized p.p.b.-range mass accuracies and proteome-wide protein quantification. Nat. Biotechnol..

[53] Tyanova S., Temu T., Cox J. (2016). The MaxQuant computational platform for mass spectrometry-based shotgun proteomics. Nat. Protoc..

[54] Cox J., Michalski A., Iepsen E.W. (2011). Software Lock Mass by Two-Dimensional Minimization of Peptide Mass Errors. J. Am. Soc. Mass Spectrom..

[55] Cox J., Hein M.Y., Luber C.A., Paron I., Nagaraj N., Mann M. (2014). Accurate proteome-wide label-free quantification by delayed normalization and maximal peptide ratio extraction, termed MaxLFQ. Mol. Cell. Proteom..

[56] Deutsch E.W., Csordas A., Sun Z., Jarnuczak A., Perez-Riverol Y., Ternent T., Campbell D.S., Bernal-Llinares M., Okuda S., Kawano S. (2017). The ProteomeXchange consortium in 2017: Supporting the cultural change in proteomics public data deposition. Nucleic Acids Res..

[57] Perez-Riverol Y., Csordas A., Bai J., Bernal-Llinares M., Hewapathirana S., Kundu D.J., Inuganti A., Griss J., Mayer G., Eisenacher M. (2019). The PRIDE database and related tools and resources in 2019: Improving support for quantification data. Nucleic Acids Res..

