# Supplementary material for: Profiling the Murine SUMO Proteome in Response to Cardiac Ischemia and Reperfusion Injury
Source: Molecules. 2020 Nov 27;25(23):5571. doi: 10.3390/molecules25235571 (PMC7731038; doi:10.3390/molecules25235571)
Supplement: Supplementary file 1 [file molecules-25-05571-s001.zip › Hotzetal_Molecules_Suppl_Materials_Revised/Hotzetal_Molecules_Revision_Suppl_final.docx]

**Profiling the murine SUMO proteome in response to cardiac ischemia and reperfusion injury**

Paul W. Hotz^1^, Marion Wiesnet^2^, Georg Tascher^1^, Thomas Braun^2^, Stefan Müller^1^*, Luca Mendler^1^*

*shared corresponding authors

**Supplementary information**

**Fig. S1** **SUMOylation and** **SENP activities change dynamically during ischemia and reperfusion in the mouse heart**

**A:** Cardiac Troponin I (cTnI) serum levels from sham, ischemic (isch), and ischemic/reperfused (I/R, reperfusion time 1hr or 24 hrs) mice by an ultra-sensitive mouse cTnI ELISA-kit (Life Diagnostics). Data are expressed in mean ± SEM, asterisks show significant differences compared to sham (n=6-9, **p<0.01, ****p<0.0001).

**B-C**: Whole homogenates (1% SDS buffer) from sham, isch, I/R1 and I/R24 left ventricles (LV) of mouse heart were separated by pre-casted 4-20% gradient gels (Bio-Rad). Immunoblotting was performed by anti-SUMO1 (**B**) and anti-SUMO2 antibodies (**C**). Blots are overexposed (SUMO1: 1 min, SUMO2: 3 min) so that lower molecular weight signals are also visible. Mouse IgGs (heavy and light chains) are cross-reacting with anti-mouse secondary antibodies.

**D-E**: SUMO protease activities in sham, isch, I/R1 and I/R24 LV heart lysates in SEM buffer was determined by preincubating the lysates for 15 min at 25°C with either HA-SUMO1-VS (**D**) or HA-SUMO2-VS (**E**) probes followed by separation of the homogenates by SDS-PAGE. Western blotting was performed by adding anti-HA antibody. HA signal gives distinct bands representing enzymatically active SUMO-protease adducts bound to either HA-SUMO1-VS (**D**) or HA-SUMO2-VS-probes (**E**). The most active SUMO protease forms preferentially an adduct with SUMO2-VS at about 95 kD in LV homogenates (**E**).

**F:** SENP3 activities in sham, isch, I/R1 and I/R24 LV heart lysates in SEM buffer was determined as in **D** by preincubating the lysates for 15 min at 25°C with HA-SUMO1-VS probes followed by separation of the homogenates by SDS-PAGE. Western blotting was performed by adding anti-SENP3 antibody. As for negative control, homogenates were treated with NEM (10 mM) to inhibit cysteine protease activity (first lane). Vinculin was used as loading control. Quantification of the respective blot normalized to vinculin is shown. Data are expressed in mean ± SEM, asterisks show significant differences compared to sham (n=3, *p<0.05, **p<0.01).

**Fig. S2** **Enriched and exclusive SUMO1 target proteins in cardiac I/R injury**


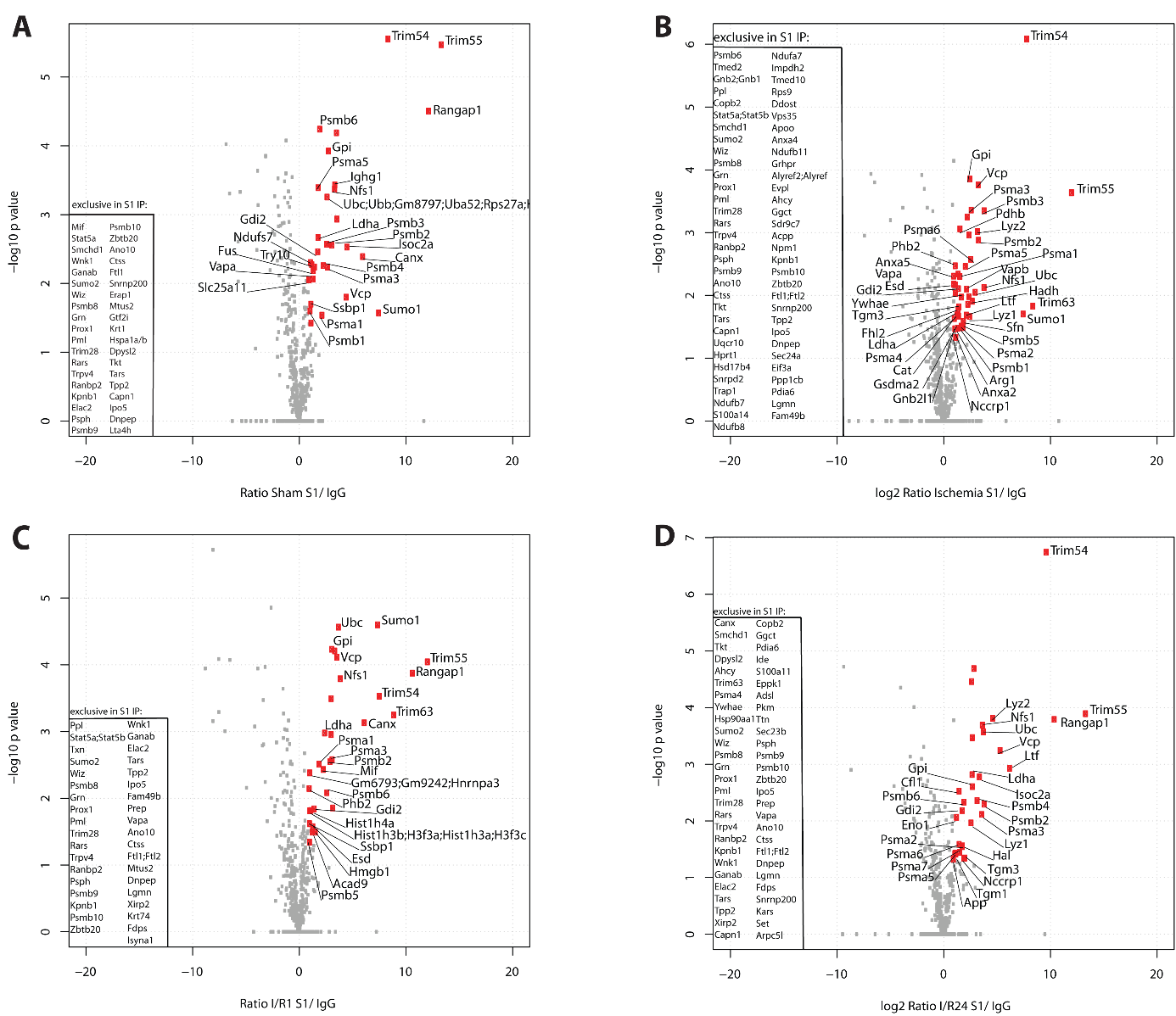


Volcano plots summarizing the results of SUMO1 IP performed from mouse hearts in sham (**A**) ischemic (ischemia, **B**), 1h-reperfused (I/R1, **C**) and 24h-reperfused hearts (I/R24, **D**). Protein target hits were assessed by a Student’s t test comparing LFQ intensities of SUMO1 IPs and IgG control IPs from the same heart. The difference of the log2 mean LFQ intensities between SUMO1 and IgG IPs was plotted against the negative logarithmized p values. Proteins with 1,5-fold higher LFQ intensity in SUMO1 IP- compared to IgG IP and a p value < 0.05 were considered as high-confidence SUMO1 target proteins and are indicated by red dots. Proteins that were exclusively detected in at least two out of three SUMO1 IPs (but not in IgG IPs) are shown in the box on the left.

**Fig. S3** **Enriched and exclusive SUMO2/3 target proteins in cardiac I/R injury**


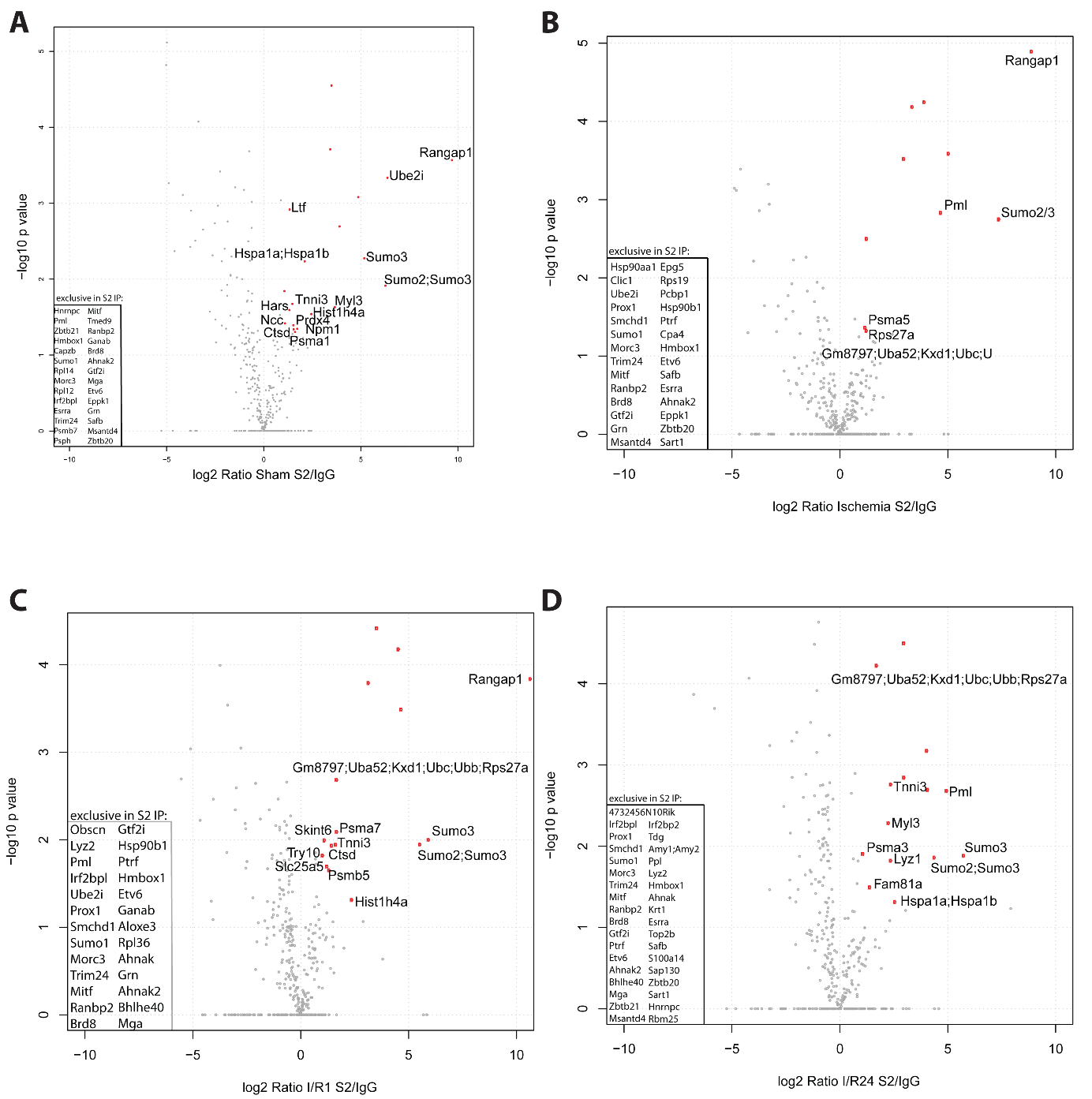


Volcano plots summarizing the results of SUMO2/3 IP performed from mouse hearts in sham (**A**) ischemic (isch, **B**), 1h-reperfused (I/R1, **C**) and 24h-reperfused hearts (I/R24, **D**). Protein target hits were assessed by a Student’s t test comparing LFQ intensities of SUMO2/3 IPs and IgG control IPs from the same heart. The difference of the log2 mean LFQ intensities between SUMO2/3 and IgG IPs was plotted against the negative logarithmized p values. Proteins with 1,5-fold higher LFQ intensity in SUMO2/3 IP- compared to IgG IP and a p value < 0.05 were considered as high-confidence SUMO2/3 target proteins and are indicated by red dots. Proteins that were exclusively detected in at least two out of three SUMO2/3 IPs (but not in IgG IPs) are shown in the box on the left.

**Fig. S4 Significantly changed SUMO1 target proteins in cardiac I/R injury**


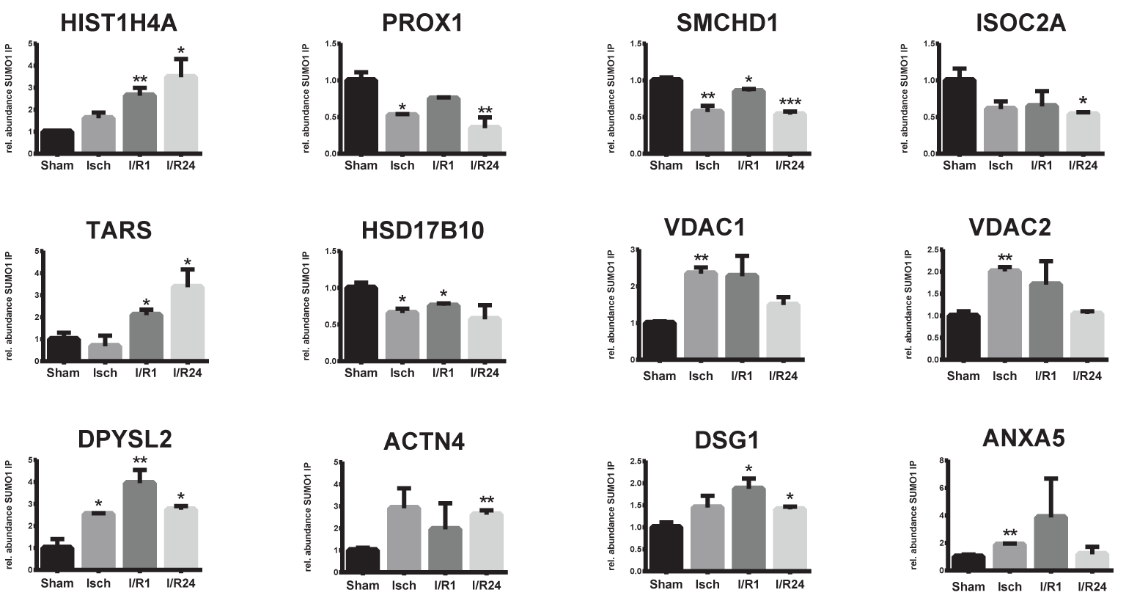


Bar diagrams of significantly changed SUMO1 targets in experimental conditions sham, isch, I/R1, I/R24. Data are expressed in mean ± SEM, asterisks show significant differences compared to sham (n=3, *p<0.05, **p<0.01).

**Fig. S5 Significantly changed SUMO2/3 target proteins in cardiac I/R injury**


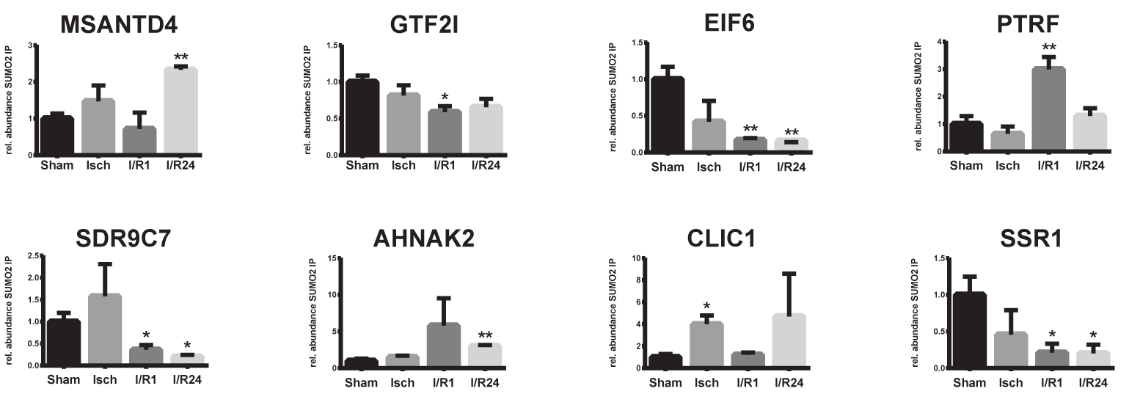


Bar diagrams of significantly changed SUMO2/3 targets in experimental conditions sham, isch, I/R1, I/R24. Data are expressed in mean ± SEM, asterisks show significant differences compared to sham (n=3, *p<0.05, **p<0.01)

**Supplementary Tables**


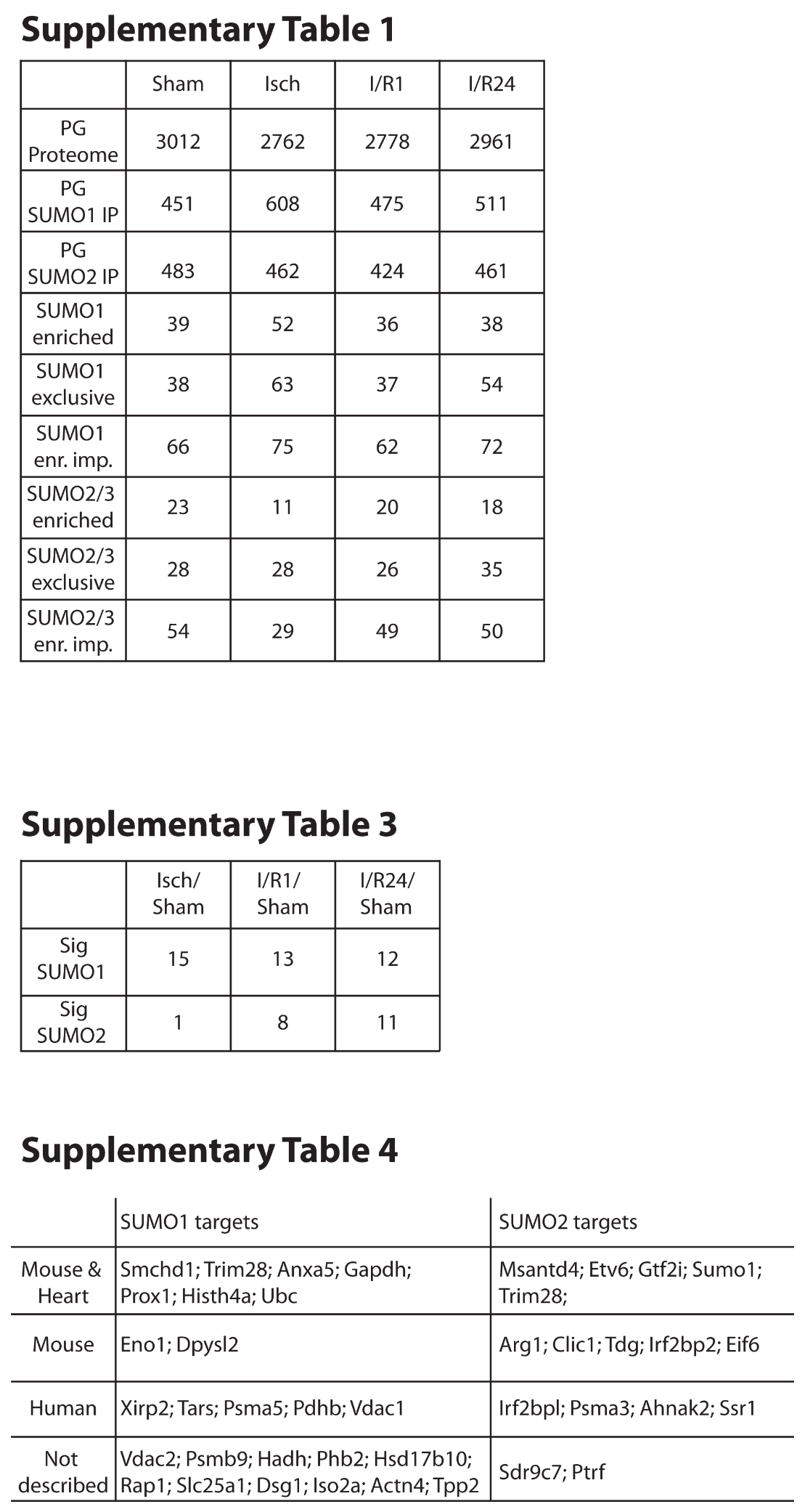


**Suppl. Table 1** Total number of peptides and protein groups (PG) identified in proteome, SUMO1 and SUMO2/3 immunoprecipitation (IP) in different experimental conditions. Sham: sham operated, Isch: 30min ischemia, I/R1: 30min ischemia followed by 1h reperfusion, I/R24: 30min ischemia followed by 24h reperfusion. Proteins with 1,5-fold higher LFQ intensity in SUMO IP- compared to IgG IP and a p value < 0.05 were considered as **enriched** SUMO targets. Proteins that were exclusively detected in at least two out of three SUMO IPs (but not in IgG IPs) were considered as **exclusive** SUMO targets. After imputation of missing values, the numbers of significantly enriched **imputated** targets (enr. imp.) are also shown.

**Suppl. Table 2** STRING analysis of the imputated SUMO1 and SUMO2 target proteins in the murine heart in response to ischemia and reperfusion. Kategories: BP: biological process, MF: molecular function, CC: cellular compartment, Pfam: Protein families

**Suppl. Table 3** Total number of significantly regulated protein groups in proteome, SUMO1 and SUMO2 immunoprecipitation (IP) in different experimental conditions compared to sham. Sham: sham operated, Isch: 30 min ischemia, I/R1: 30 min ischemia followed by 1h reperfusion, I/R24: 30 min ischemia followed by 24h reperfusion. Sig: significantly regulated. In proteome, the number of significantly changed protein groups undergoing more than 20% alteration (up/down) are shown.

**Suppl. Table 4** Significantly regulated SUMO1 and SUMO2 target proteins in this study compared with those in previous studies in murine wild type heart, murine tissues/cells, human tissue/cells or not described yet (^42^, ^43^).
